# Supplementary material for: Correlation of the High-Resolution Computed Tomography Patterns of Intrathoracic Sarcoidosis with Serum Levels of SAA, CA 15.3, SP-D, and Other Biomarkers of Interstitial Lung Disease
Source: Int J Mol Sci. 2023 Jun 28;24(13):10794. doi: 10.3390/ijms241310794 (PMC10341825; doi:10.3390/ijms241310794)
Supplement: Supplementary file 1 [file ijms-24-10794-s001.zip › ijms-2432187-supplementary.pdf]

Supplementary material

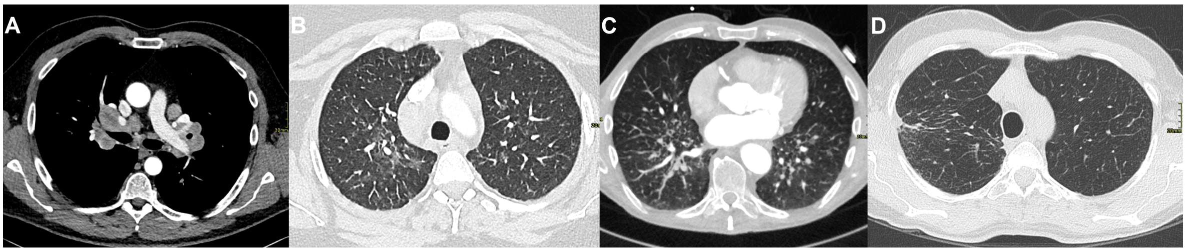

Figure S1: HRCT characteristics of sarcoidosis.

A: contrast enhanced chest CT - mediastinal and bilateral hilar lymphadenopathy, B: HRCT of the chest - sarcoidosis with predominant ground glass pattern, C: HRCT of the chest - sarcoidosis with perilymphatic and peribronchovascular involvement, D: HRCT of the chest - sarcoidosis with signs of fibrotic pulmonary changes

HRCT: high resolution computed tomography.
